# Therapeutic Use of Vitamin C in Cancer: Physiological Considerations

**DOI:** 10.3389/fphar.2020.00211

**Published:** 2020-03-03

**Authors:** Francisco J. Roa, Eduardo Peña, Marcell Gatica, Kathleen Escobar-Acuña, Paulina Saavedra, Mafalda Maldonado, Magdalena E. Cuevas, Gustavo Moraga-Cid, Coralia I. Rivas, Carola Muñoz-Montesino

**Affiliations:** ^1^Departamento de Fisiopatología, Facultad de Ciencias Biológicas, Universidad de Concepción, Concepción, Chile; ^2^Departamento de Fisiología, Facultad de Ciencias Biológicas, Universidad de Concepción, Concepción, Chile

**Keywords:** vitamin C, cancer therapy, cancer, SVCT2, GLUT, vitamin C transporters

## Abstract

Since the early studies of William J. McCormick in the 1950s, vitamin C has been proposed as a candidate for the treatment of cancer. A number of reports have shown that pharmacological concentrations of vitamin C selectively kill cancer cells *in vitro* and decrease the growth rates of a number of human tumor xenografts in immunodeficient mice. However, up to the date there is still doubt regarding this possible therapeutic role of vitamin C in cancer, mainly because high dose administration in cancer patients has not showed a clear antitumor activity. These apparent controversial findings highlight the fact that we lack information on the interactions that occurs between cancer cells and vitamin C, and if these transformed cells can uptake, metabolize and compartmentalize vitamin C like normal human cells do. The role of SVCTs and GLUTs transporters, which uptake the reduced form and the oxidized form of vitamin C, respectively, has been recently highlighted in the context of cancer showing that the relationship between vitamin C and cancer might be more complex than previously thought. In this review, we analyze the state of art of the effect of vitamin C on cancer cells *in vitro* and *in vivo*, and relate it to the capacity of cancer cells in acquiring, metabolize and compartmentalize this nutrient, with its implications on the potential therapeutic role of vitamin C in cancer.

## Introduction

Vitamin C is an essential nutrient for humans, acting as an antioxidant and a cofactor for several enzymatic reactions. These reactions involve dioxygenase enzymes which participate in a number of physiological processes, such as collagen synthesis, carnitine synthesis, norepinephrine and serotonin synthesis, hypoxia-inducible transcription factor (HIF) regulation and histone demethylation (Verrax and Calderon, 2008; Du et al., 2012).

Humans, unlike most mammalian species, are unable to synthesize vitamin C, hence it is an essential dietary component and humans need to acquire this vitamin from external sources, such as vegetables and fruits (Rivas et al., 2008). The recommended dose for an adult is around 100 mg per day, which has shown to maintain plasmatic concentration of 50 μM. However, when intracellular content is measured, differential concentrations of vitamin C are found depending on the tissue. Circulating leucocytes, pituitary gland, adrenal glands and brain, among others, accumulate largely higher concentrations than plasma reaching millimolar range (Rose, 1988).

Vitamin C, which performs most of its functions inside the cells, must enter through the plasma membrane of specific cells in a process that requires the participation of transporters. Vitamin C exists in two molecular forms with different chemical stability, half-life *in vivo*, and transport mechanism. The oxidized form, dehydroascorbic acid (DHA) is transported from extracellular medium into the cell by glucose transporters (GLUTs), while the reduced form, ascorbic acid (AA), is transported by sodium-vitamin C co-transporters (SVCT) (Vera et al., 1993; Tsukaguchi et al., 1999).

After more than 50 years of research focused in the role of vitamin C in cancer, it still remains unclear the ways that this nutrient specifically affects cancer cells. And, until recently, there was a lack of information on the physiological aspects of their interaction: it was practically unknown how cancer cells acquire vitamin C, and how it is metabolized or compartmentalized inside these cells.

## Vitamin C Transport and Compartmentalization in Cancer Cells

As we addressed before, the knowledge on vitamin C uptake capacity and the organelle requirements of this nutrient in the context of cancer is still incomplete. Most of what is known comes from the analysis of GLUTs, which even if they have been mainly studied in the context of glucose uptake capacities in cancer, have direct implications in DHA uptake. In this context, it has been well described that cancer tissues overexpress GLUTs, which leads to an increased capacity to acquire glucose (Adekola et al., 2012; Ancey et al., 2018).

SVCTs on the other hand have been poorly studied. Meanwhile SVCT1 does not appear to have relevance in cancer (Pena et al., 2019), different studies have proposed that SVCT2 has a major function in tumors. The first of them showed that breast tumors have higher levels of SVCT2 expression compared to normal cells (Hong et al., 2013). In fact, overexpression of this transporter led to an increased chemosensitivity to high dose of ascorbate, which resulted in augmented reactive oxygen species (ROS) production and ulterior cell death. Oppositely, siRNA against SVCT2 render the cancer cells resistant to this treatment (Hong et al., 2013). Therefore, SVCT2 might be implicated in the ascorbate induced cancer cell death phenomena. Similar results were observed by two groups in cholangiocarcinoma cells (Wang et al., 2017), hepatocellular carcinoma (Lv et al., 2018) and colon cancer cells (Cho et al., 2018), where SVCT2 expression determines the susceptibility to pharmacological ascorbate-induced cell death.

A comprehensive study on this matter, focused on breast cancer cells mechanisms to acquire vitamin C was recently published (Pena et al., 2019; Roa et al., 2019). This work has shown that most (if not all) of the vitamin C that is capture by breast cancer cells is in the oxidized form. These cells are not able to uptake AA at physiological concentrations. Interestingly, SVCT2 is expressed in cancer cells, and as it was shown before by Hong et al. (2013) its expression is restricted to breast cancer cells, but not normal breast tissue. The lack of AA transport was due to the fact that SVCT2 is absent at the cell surface, with most of the protein distributed within the inner membrane of the mitochondria. Mitochondrial SVCT2 was previously described in U937 cells (Azzolini et al., 2013) and HEK293 cells (Munoz-Montesino et al., 2014) and it was also observed in various cancer derived cell lines from different origin, where the presence of SVCT2 is always link to the mitochondria (Pena et al., 2019). Considering these data, at least under physiological conditions, the external source of vitamin C for cancer cells must be DHA and the transport mechanism implied is through GLUTs. Since DHA is not the most abundant form of vitamin C *in vivo*, Pena et al. (2019), developed an experimental system to check if AA could be oxidized locally by PMA-activated neutrophil like cells, which are producing ROS. Co-culture experiments in the presence of AA showed that cancers cells were able to acquire vitamin C in a process inhibited by cytochalasin B, a glucose transporter inhibitor, and by glucose, indicating that they were able to acquire DHA through GLUTs by bystander effect (Nualart et al., 2003). In this regard, it is important to emphasize that the main form of vitamin C found after this co-culture was AA, meaning cancer cells are able to efficiently reduce DHA once inside the cell (Pena et al., 2019).

On the other hand, the observation that a vitamin C transporter is located in an organelle membrane is not new. Other groups have reported the relevance of organelle vitamin C transporters in a number of cellular systems, including plants, mammals and other organisms (Banhegyi et al., 2014). In this context, organelle requirements for vitamin C in cancer cells have not been previously described. Therefore, the observation that the mitochondrial form of SVCT2 (mitSVCT2) is linked to cancer pathology (Pena et al., 2019), and absent in normal cells, it may have unforeseen implications: mitochondrial vitamin C could be relevant for cancer cell development or survival.

## Vitamin C in the Treatment of Cancer: an Historical Perspective

Based in the known role of vitamin C in collagen synthesis, about six decades ago, William J. McCormick hypothesized that cancer metastasis could be related to a vitamin C deficiency, due to a poor collagen formation and connective tissue degeneration. Hence, the maintenance of collagen synthesis at optimal levels by using vitamin C was proposed to limit metastasis process (McCormick, 1954, 1959). Since this first assumption until now, the possible benefit of vitamin C in the treatment of cancer has been controversial.

McCormick’s hypothesis was extended in the 1970s by Ewan Cameron et al. Cameron proposed that vitamin C inhibited the enzyme hyaluronidase, which reduces tissue disruption and cancer cell proliferation (Cameron and Rotman, 1972). For this, Cameron performed the first clinical studies analyzing the anti-cancer effect of ascorbate, initially together with Allan Campbell (Cameron and Campbell, 1974; Cameron et al., 1975) and then with the Nobel Prize winner Linus Pauling. In two retrospective studies, Cameron and Pauling suggested a therapeutic effect of high-dose vitamin C treatment in advanced cancer. Terminal cancer patients receiving intravenous (IV) followed by oral doses of ascorbate, showed increased survival times and symptomatic relief compared to control patients (Cameron and Pauling, 1976, 1978). These studies created a controversy and the results were criticized, among other things, for the lack of blinding inherent to a retrospective trial and the possibility of a placebo effect.

Studies performed at the Mayo Clinic tried to evaluate Cameron and Pauling’s results, analyzing the efficacy of vitamin C in randomized double-blind placebo-controlled trials. Prospective studies from Creagan et al. (1979) and Moertel et al. (1985) found no effect of oral ascorbate treatment in the survival of patients with advanced cancer, compared to patients receiving placebo. These results silenced for many years the initial attempt to determine the use of vitamin C as an anti-cancer agent.

None of the above-mentioned studies measured plasmatic levels of AA, a fundamental issue to understand the real effect of the vitamin C administration in patients. This was clarified in studies performed by the National Institute of Health (NIH), in order to establish dietary recommendations for AA (Levine et al., 1996, 2001). Physiological analyses in healthy young subjects showed that ascorbic acid pharmacokinetics differ depending on the way of administration. When subjects received oral doses, low plasma ascorbate concentrations were achieved (around 100–200 μM), while IV doses resulted in concentrations 100-fold higher than oral (around 15 mM) (Padayatty et al., 2004). This is a consequence of a limited intestinal absorption, renal reabsorption and excretion in oral administration. Unlike oral, IV administration eludes this tight control and produces high plasmatic concentrations that are safe or tolerable for humans (Padayatty et al., 2004).

Thus, high (“pharmacologic”) concentrations of AA are achieved only with IV, not with oral administration (“physiologic”). Based in this evidence, it was proposed that only pharmacologic ascorbate concentrations could act as a drug, and the interest for using vitamin C as an anti-cancer agent reemerged.

## Effect of Pharmacologic Vitamin C in Cancer Cells

Since the basic knowledge regarding vitamin C pharmacokinetics was established, several studies analyzing the effect of pharmacologic ascorbate in cancer cells have been reported. Initially, *in vitro* studies in a number of human and mice cancer cell lines showed that ascorbic acid at concentrations around 20 mM selectively kill cancer cells, without effect in normal cell lines. In addition, the authors proposed that the cancer cell death inducing mechanism was dependent on hydrogen peroxide (H_2_O_2_) formation with ascorbate radical as an intermediate (Chen et al., 2005). The same research group later confirmed in rats that ascorbic acid at pharmacologic levels, achieved by IV or parenteral administration, induced ascorbate radical and H_2_O_2_ formation in the extracellular medium (Chen et al., 2007).

Regarding *in vivo* studies, Chen et al. (2008) showed that intraperitoneal administration of pharmacologic ascorbate decreased growth rates of human ovarian, mouse pancreatic and rat glioblastoma tumors causing a prooxidant effect. Similarly, Verrax and Calderon (2009) showed that intraperitoneal administration of ascorbate decreased the growth rate of a murine hepatoma in mice. The mechanism of cytotoxicity is linked to the production of extracellular H_2_O_2_ and involves intracellular transition metals (Chen et al., 2008; Verrax and Calderon, 2009). In the same line, several reports support the induction of ROS achieved by high concentrations of vitamin C in cancer cells as a mechanism for cancer cell death induction: in human pancreatic tumor (Du et al., 2010), in human mesothelioma (Takemura et al., 2010), in human breast cancer (Hong et al., 2013), among others.

Experiments performed *in vitro* to test compatibility with other anti-carcinogenic substances revealed that AA can have a synergistic effect with some of them (Espey et al., 2011; Ma et al., 2014; Hatem et al., 2018; O’Leary et al., 2018; Graczyk-Jarzynka et al., 2019). For instance, Gemcitabine in combination with AA (Espey et al., 2011) have a synergistic cytotoxic effect in eight pancreatic cancer cell lines, which is mediated by the pro-oxidant effect of ascorbate, again with an increase in the production of H_2_O_2_. In addition, mice bearing pancreatic tumor xenografts showed a higher inhibition in tumor growth when treated with the mixture of Gemcitabine and AA, compared to mice treated only with the drug (Espey et al., 2011). A synergistic effect of AA and two of the chemotherapeutic drugs used in the treatment of ovarian cancer was also observed: carboplatin and paclitaxel, which inhibited tumor growth in models of mice with ovarian cancer and decreased the adverse effects of chemotherapy in patients with this disease. In triple negative breast cancer (TNBC), a new combination with AA was tested using Auranofin (AUF), which targets thioredoxin reductase (TRXR) (Hatem et al., 2018). In combination, these molecules also act in a synergistic way, inducing extracellular production of H_2_O_2_ and cytotoxicity against MDA-MB-231 (a breast cancer derived cell line) in cell culture and in xenografts in mice. Proteomic and functional analyses in this model suggested that prostaglandin reductase 1 expression was linked with the breast cancer sensitivity to AUF/AA combination (Hatem et al., 2018).

The synergistic effect of ascorbate in the treatment of various types of cancer has been observed not only in combination with chemotherapeutic drugs but also in treatments with ionizing radiation (Du et al., 2015). *In vitro*, AA has been shown to potentiate the cytotoxic effect of ionizing radiation in various pancreatic cancer cell lines, but not in non-tumorigenic cell lines. This synergistic effect is attributed to an increase in oxidative stress within the tumor cells, which causes substantial oxidative damage. Murine models of pancreatic cancer, subjected to both radiotherapy and AA treatment showed a reduction in tumor growth without observing damage to their gastrointestinal system, with an increase in survival time. It can be proposed that AA can be used as a complementary therapy to radiotherapy in patients with pancreatic cancer (Du et al., 2015).

Vitamin C toxicity could also be associated with its oxidation byproducts. Lu et al. (2018) reported that DHA, which is transporter by GLUTs and no SVCTs enhance the efficacy of oxaliplatin through redox modulation in a gastric cancer model (Lu et al., 2018). The question whether AA or DHA is the most cytotoxic vitamin C species in cancer was addressed recently in breast cancer derived cell lines. AA showed higher toxicity in TNBC cells than DHA, increasing H_2_O_2_ in the extracellular medium and in intracellular compartments (El Banna et al., 2019).

AA antitumoral effect is not only restricted to the promotion of ROS. Ge et al. (2018) suggests that vitamin C analogs may play a role in cell reprogramming and growth regulation by enhancing the catalytic activity of ten-eleven translocation (TET) enzymes responsible for the oxidation of 5-methylcytosine (5 mC) a well-known differentiation promoting agent. Loss of 5 mC expression correlates with the development of several aggressive form of cancer. Therefore, AA dependent restoration underlines the role of AA and its analogs in cancer epigenetics, inducing an alteration in the differentiation potential of cancer stem cells, playing a role avoiding cancer metastasis (Ge et al., 2018; Satheesh et al., 2020).

Undoubtedly, the data in the last 15 years showing an anti-cancer effect of high doses of AA represent an interesting opportunity for the field of therapeutic use of vitamin C. However, as described below, these results have not been clearly replicated in humans. An important reason is the difference in vitamin C metabolism between mouse models and human. While humans need to acquire vitamin C from the diet, mice synthesize its own. This essential difference should be considered when interpreting the results from xenograft models.

## Vitamin C Administration in Clinical Studies

Since the early 2000s, several studies including case reports and clinical trials, have been analyzed the effect of IV vitamin C in patients with different types of cancer. Two initial reports showed that treatment with high-dose IV ascorbate is well tolerated for cancer patients (Padayatty et al., 2006; Hoffer et al., 2008). However, although one study analyzing three cases showed long survival times of patients (Padayatty et al., 2006), the second study analyzing 24 cases failed to demonstrate anticancer activity of vitamin C (Hoffer et al., 2008). A study considering 125 breast cancer patients showed that IV ascorbate reduces chemotherapy-related side effects, such as nausea, fatigue and dizziness (Vollbracht et al., 2011). Similar results were obtained in a study with 60 patients with different types of cancer, where IV ascorbate improved their quality of life (Takahashi et al., 2012). In addition, vitamin C administrated alone also improved quality of life in a study including 17 patients with different solid tumors, although no patient showed an objective antitumor response (Stephenson et al., 2013).

Two studies evaluated the effect of IV ascorbate in the survival of patients with stage IV pancreatic cancer under standard chemotherapy treatment. Both studies reported reduction of tumor mass and possible improvements of survival in 14 (Monti et al., 2012) and nine patients (Welsh et al., 2013) analyzed. Another study analyzed 14 patients with different types of cancer receiving chemotherapy, and reported that IV ascorbate administration produces an increased energy and functional improvement in patients (Hoffer et al., 2015). A randomized controlled trial performed in 27 patients with ovarian cancer showed that IV ascorbate treatment reduced the chemotherapy-associated toxicity, however, minimal effect on survival of patients was observed (Ma et al., 2014).

A study performed in 23 patients with metastatic castration-resistant prostate cancer showed that IV ascorbate administration did not result in disease remission or anticancer effect (Nielsen et al., 2017). On the other hand, a study analyzing 13 glioblastoma patients receiving radiotherapy and 14 non-small-cell lung cancer patients receiving chemotherapy, showed that IV ascorbate treatment extended survival of patients (Schoenfeld et al., 2017). Similarly, a report analyzing 14 pancreatic cancer patients receiving IV ascorbate and chemotherapy showed the possibility to prolong survival of patients (Polireddy et al., 2017). Another report performed in 73 patients with acute myeloid leukemia showed that IV ascorbate treatment combined with chemotherapy produced a higher complete remission and prolonged survival compared to patients who received only chemotherapy (Zhao et al., 2018). In a retrospective study in 613 hepatocellular carcinoma patients, it was shown that IV ascorbate administration improved disease-free survival of patients after surgery (Lv et al., 2018). Finally, a phase I metastatic colorectal and gastric cancer study in 36 patients applying high-dose AA in combination with mFOLFOX6 or FOLFIRI, showed potential clinical efficacy in these patients (Wang et al., 2019).

In general, most of the studies described above reported that vitamin C treatment could improve quality of life or reduce chemotherapy-related side effects in cancer patients. However, only a few works showed a clear antitumor effect of vitamin C or the consequent prolongation of patients’ survival, especially when vitamin C is applied as an adjuvant. The differential results could be due to the disparities between the studies, such as doses, number of patients, types of cancer, methodologies, and parameters studied, issue that has been previously analyzed by several reviews (Fritz et al., 2014; Jacobs et al., 2015; Carr and Cook, 2018; Nauman et al., 2018). Even more important, components involved in cell transport and compartmentalization of vitamin C should be considered in each particular case to interpret these clinical results and understand the role of vitamin C in cancer.

## The Cancer-Vitamin C Paradox: Physiological Considerations

In this review, we have briefly gathered together more than 6 decades of studies focused on unraveling the relationship between vitamin C and cancer. Contradictory effects of this nutrient in cancer have been reported: on one hand the evidence showing anti-cancer activity with differential results depending on a number of factors; and, on the other hand, some evidence from *in vitro* studies showing that certain cancer cells exposed to vitamin C inhibited apoptosis or DNA damage (Perez-Cruz et al., 2007; Heaney et al., 2008).

The first important element to consider when analyzing cancer-vitamin C connection is the administration route, which has been well established in humans: limited by the intestinal barrier, oral administration, no matter the dose, in plasma will reach only in the μM range of concentration. Therefore, to achieve “pharmacological” or therapeutic plasmatic concentrations in the mM range, intravenous administration is required. The effect that vitamin C has on tumor cells will also depend on the dose. Meanwhile physiological doses do not induce cell death, pharmacological doses are able to specifically kill cancer cells *in vitro* and in xenograft mice *in vivo*.

**FIGURE 1:**
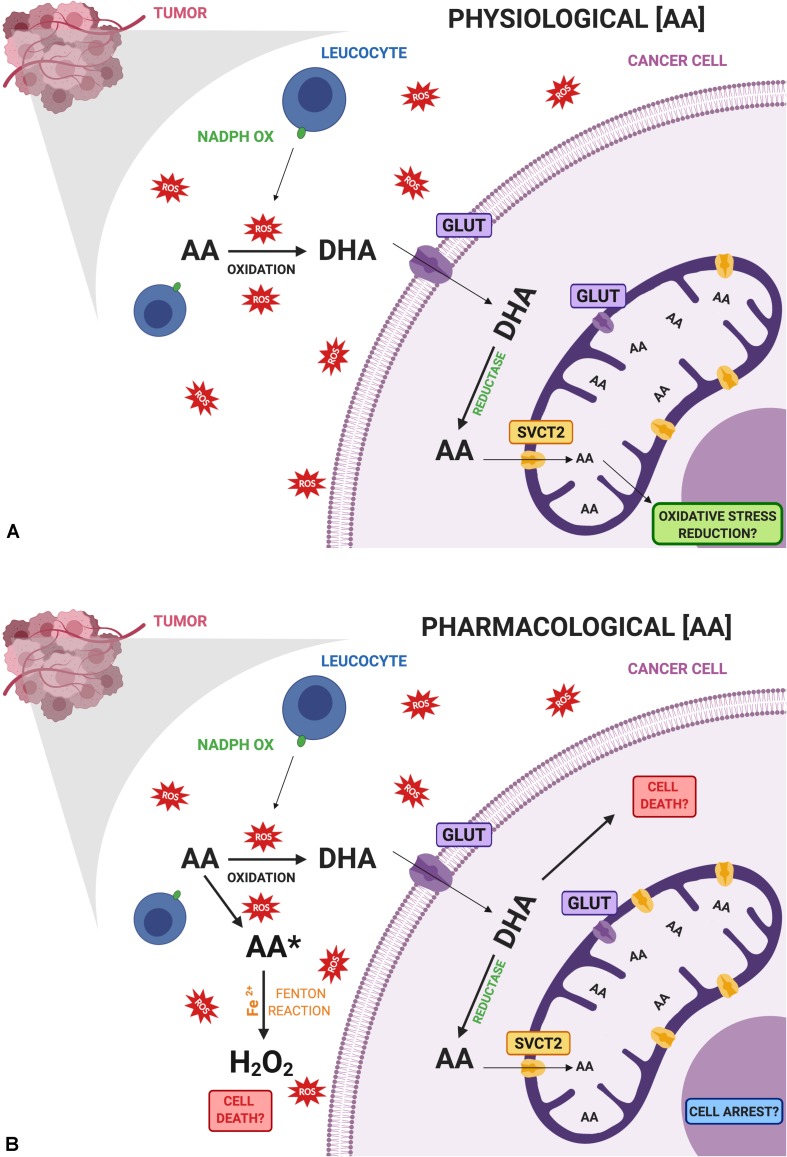
Comparative diagram of vitamin C doses and its possible effects on cancer cells. **(A)** Physiological doses of ascorbic acid (AA) are oxidized with the help of activated leucocytes that are infiltrating the tumor, producing dehydroascorbic acid (DHA), which is the main form transported by cancer cells. Once inside the cell, DHA is rapidly reduced to AA where it can enter mitochondria via SVCT2 reducing oxidative stress at this level. **(B)** Pharmacological doses of AA may induce the production of ascorbate radical (AA*) and hydrogen peroxide (H_2_O_2_) inducing cell death. On the other hand, increased amounts of DHA entering cancer cells through GLUTs may participate in cell death due to an increase in oxidative stress. Role of mitochondrial GLUTs must be further studied in cancer. Finally, intracellular vitamin C could contribute to cell arrest. All these events are related to the cytotoxic effects of vitamin C in cancer cells. Created with BioRender.com.

The second consideration is regarding the specific abilities of each cancer to acquire this nutrient and metabolize it (Figure 1). The role of SVCT2 and GLUTs, partly addressed by the studies performed so far, must be studied in each case in particular, considering their intracellular distribution. In this context, breast cancer cell lines have shown a strong capacity to acquire only the oxidized form of vitamin C and an efficient machinery to reduce it to AA. Even more, they overexpress mitochondrial SVCT2 that could be related to oxidative stress resistance. Nevertheless, when studied in pharmacological doses, the same or similar cancer cell lines showed to be sensitive to vitamin C treatment. We proposed a model considering the vitamin C transporters present in breast cancer cells, and how this can affect their capacity to interact with vitamin C (Figure 1). At physiological doses (Figure 1A), DHA byproduct from AA could enter the cancer cell, be reduced and enter the mitochondria, which could be related with the protective effect of vitamin C. However, at pharmacological doses (Figure 1B), extracellular accumulation of AA is able to induce H_2_O_2_ production, being one possible mechanism inducing cancer cell death. In addition, the direct effect of an increased DHA uptake by cancer cells when high concentrations of AA are available must be studied. Since cancer cells efficiently reduce DHA, this might be another mechanism that could promote cell death by glutathione depletion. Finally, vitamin C uptake capacity is directly influencing its epigenetic role that has relevance avoiding metastasis.

In conclusion, the knowledge on the capacities of cancer cells in acquiring and compartmentalize vitamin C is crucial and has direct implications for rational development of new vitamin C intervention procedures in human cancer, with plasma membrane glucose/dehydroascorbic acid transporters and mitochondrial ascorbic acid transporters as novel primary targets.

## Author Contributions

EP, FR, GM-C, CR, and CM-M: conceptualization. KE-A and PS: figure design. GM-C and CR: funding acquisition. FR, EP, MM, MG, MC, and CM-M: article reviewing. FR, GM-C, CR, and CM-M: resources, supervision, and writing – review and editing. FR, EP, MG, MC, MM, GM-C, CR, and CM-M: writing – original draft.

## Conflict of Interest

The authors declare that the research was conducted in the absence of any commercial or financial relationships that could be construed as a potential conflict of interest.
